# Supplementary material for: Patterns of Intron Gain and Loss in Fungi
Source: PLoS Biol. 2004 Nov 30;2(12):e422. doi: 10.1371/journal.pbio.0020422 (PMC532390; doi:10.1371/journal.pbio.0020422)
Supplement: Table S1 — Also available at http://genes.mit.edu/NielsenEtAl/. (4.3 MB ZIP). [file pbio.0020422.st001.zip › NielsenEtAl/html/1194.html]

AN8122.1.NCU00754.1.MG04158.1.FG09697.1


```
 CLUSTAL W (1.82) Multiple Sequence Alignments - Introns Inserted


Sequence 1: MG04158.1	617 aa
Sequence 2: FG09697.1	612 aa
Sequence 3: AN8122.1	521 aa
Sequence 4: NCU00754.1	586 aa
Alignment Length: 628 aa
Number Identitical Residues: 303 aa
Alignment Score (without introns) 14688


MG04158.1 	MAEKIDLKSSPEKGQAEENSVGSTQQHPTISSANPKAESGTSLHRLSHATPTSEHL~PPT
NCU00754.1	MAITIRKDCPPQWRQHLSQPIG----------HFHWLVLGHAVGRQKRTARYAR-L1APT
FG09697.1 	----MDQPNSSRSSTTLENGT-----HPDEATFAPLNTHQSTMSRVRRSLSHSHSR~DGS
AN8122.1  	------------------------------------------------MGSNRGEV~DPT
          	                                                           :

MG04158.1 	ANTHSPQQVGFDPQPAVTLPYRTLNSTANIDEYVTEAPSGEIDGPPEND-----------
NCU00754.1	-------------------PRDIAMATTQAAPTAAGAPQPPKEKIPEGGE----------
FG09697.1 	QKEHR------DPDLDIDLPYRTLSANANLDEYRVEVPGGAIPGPVQPEEIARKSTNHEP
AN8122.1  	-----------------DLPYRTLTADANIEEYVQETQTGEIAKPVRTAD----------
          	                   *     : ::       .         .  .          

MG04158.1 	GSGRRYKLVTFLPDDPENPKNWSKAFKWYVTMVVALTCFVVALASSVITADIAGVVEEFG
NCU00754.1	-IQQDYKLVTFAEGDPGNPKNWSKGYKWYCTMVVAITCFVVAFCSSVITADVDSVAHEFG
FG09697.1 	GTERRYKLVTFTPGDPENPKNWSKAYKWWCTMCVALTCFVVAFCSSVITADIVGVTNDLN
AN8122.1  	GRTEDYKLVTFKVDDPENPKNWPKWKKWYVTMVVAFTCFVVAFASSVITADIEGPAEEFG
          	.  . ******  .** *****.*  **: ** **:******:.*******: . ..::.

MG04158.1 	VSEEVALLSITVFVIGFGIG1PMIFAPLSEVYGRRIIY2GSTLLVAVIFIIPCAVAQNIE
NCU00754.1	ISHEAALLPITVFVVGFGVG1PMVFAPLSEVYGRQIIY~ATTLLLAVVFIIPCAVSKNIG
FG09697.1 	VSNELALASISLFVVGFGVG~PMAFAPLSEIYGRRIIY~GSTLFIAFIFIIPCAVAKNIE
AN8122.1  	VSREVSLVVVTVFVIGFGVG1PMAFAPLSEMYGRRPVY~ALTLLLAVVFVIPCAVAENIG
          	:*.* :*  :::**:***:* ** ******:***: :* . **::*.:*:*****::** 

MG04158.1 	TLLVCRAIDGIAFSAPMTLVGGTLADLWRNEERGVPMAAFSAAPFIGPAI1GPLVGGYLS
NCU00754.1	TLIVCRLIDGIAFSAPMTLVGGTLADLWRNEERGVPMAAFSAAPFIGPAI1GPLVGGWLC
FG09697.1 	TLLVCRAIDGIAFSAPMTLVGGTLADMWRNEERGVPMAAFSAAPFIGPAI1GPLVGGFLS
AN8122.1  	TLIVCRAIDGIAFSAPMTLVGGTLADMWRNEERGVPMAAFSASPFIGPAI~GPLAGGFLA
          	**:*** *******************:***************:******* ***.**:*.

MG04158.1 	DATGWRWLYWIQLIVSFVVWVLITFTVPETYAPTILARRARQIRKKTGEQDHVTEQDLDL
NCU00754.1	LAG-WRWLYWIQLILAFVVWVLISFTVKETYAPTILGRRAAKLRKQTGDNSYVSEGDLDQ
FG09697.1 	DAAGWRWLYWIQLILAFVVWILITFTVPETYAPTILARRARKLRAETGETDHVTEQELDL
AN8122.1  	DASGWRWLYWLTLILSFVAWVLITFTVPETSAPAILARRAKKLRKSENDPKYVTETELDA
          	 * .******: **::**.*:**:*** ** **:**.*** ::* . .: .:*:* :** 

MG04158.1 	RPASERLAIFLIRPFQLLFGELIVFLISIYMSVLYGLLYM2FFVAFPIIYQEGKGYSAGT
NCU00754.1	RPFSERMSVFLIRPFQLLFRELIVLLISLYMSVLYGLLYM~FFVAYPVVFEGGKGYSHGQ
FG09697.1 	RPISERLRIFLIRPFQLLFGELIVCLISLYMSVLYGLLYM~FFVAYPIVYQKGKGYSAGK
AN8122.1  	RPIGERLRVFLLRPFQLLFLEPIVLFISLYMSVLYGLLYM~FFVAYPIVYEGGKGWSAGT
          	** .**: :**:******* * ** :**:*********** ****:*:::: ***:* * 

MG04158.1 	TGLMFIPCAVG0VILSAFCAPFVNKNYMNQVRKHNGKPPAEVRLIPMMCSCWFIPIGLFI
NCU00754.1	TGLMFIPLAVG~VICSALCAPLVNKHYLTQVQKYNGKPPAEIRLIPMMVSCWFIPIGLFI
FG09697.1 	TGLMFIPIAVG~VLMSAFCAPFVNKHYLTLVKKHNGNPPAEVRLIPMMISCWCIPIGLFI
AN8122.1  	TGLMFIPLAIG~VLLSAACAPFVNNHYLSLYSKYGGKPPAESRLIPMMISCWFIPIGLFI
          	******* *:* *: ** ***:**::*:.   *:.*:**** ****** *** *******

MG04158.1 	FAWTSYKELSWAGPAMGGFSVGFGFIFLYNSANNYLV~DSYQHQAASALAAKTFIRSFWG
NCU00754.1	FAWTSYPRLIWVGPCLAGFPIGFGFIFLYNSANNYLV~DSYQHQAASALAAKTFIRSFWG
FG09697.1 	FAWTSYSDLSWAGPAMGGFPVGFGFIFLYNSANNYLV~DSYQHQAASALAAKTCIRSFWG
AN8122.1  	FAWTSYPNIHWFGPMIGGFPVGFGFIFLYNSANNYLV1DTYQHQAASALAAKTFIRSLWG
          	******  : * ** :.**.:**************** *:************* ***:**

MG04158.1 	AAVVLFTSQMYDRLGNQWASTLLAFISVACCAIPFLFWRYGARIRRRSKYAYAGDDDDDA
NCU00754.1	AGTVLFTEQMYHRMGDQWASTFLAFLGLLCCLIPFGFWKFGARIRQRSKYAFGGDDEDGS
FG09697.1 	AAVVLFTEQMYERLGDQWASSLLAFLGLACCAIPFMFWKYGAKIRERSKYAYAGEDESVD
AN8122.1  	ASTVLFTEQMYDRLGDQWASSLLAFLALACCAIPYVFYVKGAAIRRYSRYAFADDEEALA
          	*..****.***.*:*:****::***:.: ** **: *:  ** **. *:**:..:::   

MG04158.1 	QNDEKSSVDLEKGHNKDPERDMEDLRRAQSYVSNP
NCU00754.1	QTSDLENQKDVVDPNAPLVHDSLALERARSYVSNP
FG09697.1 	DIEKVKSHNASSGQVRN-DSDDEDLRRARSYVSNP
AN8122.1  	TKA--------------------------------
          	
```
